# Supplementary material for: The relationship between latent inhibition, divergent thinking, and eyewitness memory: A study on attention to irrelevant stimuli
Source: PLoS One. 2025 Jun 23;20(6):e0315158. doi: 10.1371/journal.pone.0315158 (PMC12184939; doi:10.1371/journal.pone.0315158)
Supplement: Attachment — Submitted filename: Response letter to PlosOne.pdf [file pone.0315158.s003.pdf]

Dear Dr. Treviño Villegas,

We are grateful for the time and effort devoted to reviewing our manuscript titled *The Relationship between Latent Inhibition, Divergent Thinking, and Eyewitness Memory: A Study on Attention to Irrelevant Stimuli* (manuscript ID: PONE-D-24-53417R1). We were pleased to see that Reviewer 1 considered all previous comments to have been adequately addressed and recommended the manuscript for publication.

Regarding Reviewer 3's report, we respectfully submit that the concerns raised are already fully addressed in the current version of the manuscript. After carefully reviewing the six points raised, we found that each of them corresponds to content that is explicitly discussed in the Introduction, Method, Results, or Discussion sections. In fact, we believe that modifying the manuscript to restate these points would be redundant and risk making the text less clear or unnecessarily repetitive.

We would certainly be happy to revise the manuscript further if there were a constructive way to address the reviewer's concerns more directly. However, at present, we are not sure how to respond more proactively without reiterating content that is already there. If, as the Academic Editor, you consider that any of the issues raised by Reviewer 3 should nevertheless be explicitly reiterated or emphasized in the manuscript, we would of course be willing to revise the text accordingly.

We provide below a point-by-point response to Reviewer 3, indicating where in the manuscript each of the issues is already covered. We hope that this clarifies the coherence and soundness of our study and confirms that the comments raised do not reflect substantive flaws in the work.

We remain at your disposal should you require any further clarification.

Kind regards,

Gabriel Rodríguez
On behalf of all authors

Response to Reviewer 3

We thank Reviewer 3 for their comments. However, we respectfully note that all six concerns raised in their review are already addressed in detail in the current version of the manuscript. We provide a point-by-point response below.

(1) "The purpose of this study is not very clear, what deficiencies in this field at present? What are the problems need address?"

We respectfully disagree. The Introduction section (pp. 3–6) clearly outlines the motivation for the study, including the unresolved issue of whether attenuated latent inhibition can be a valid marker of leaky attention and how this relates to the processing of peripheral

information in eyewitness memory. The limitations of previous research using masked pre-exposure procedures are also discussed in detail (p. 6), and our methodological improvement is clearly justified.

(2) "This study used two dependent variables, but what's the relationship between them? The overall logic of the research needs to be strengthened."

The relationship between the two tasks (divergent thinking and eyewitness memory) is explicitly discussed both in the Introduction and the Discussion (pp. 5–6 and pp. 18–20), where we explain how both types of tasks are theoretically linked to the processing of irrelevant or peripheral information. We also provide clear rationale for analyzing them together in the context of individual differences in attention.

(3) "Although the divergent thinking of three dimensions is very high, the natures of the dimensions are different, the author is not recommended to integrate them."

We are aware of the differences among fluency, flexibility, and originality, and this is acknowledged in the manuscript (p. 14). For this reason, we report descriptive statistics and intercorrelations for each dimension separately (Table 1), and only compute the composite DIV score for the purpose of certain correlational and regression analyses. Importantly, we also report the separate contributions of each dimension where appropriate (p. 15–16), ensuring transparency and granularity in our analyses.

(4) "The manipulation could be further checked; the manipulation of failure data should be cleaned."

The manipulation is robust and clearly described. We explain in detail how latent inhibition was operationalized and verified through the analysis of the batting success ratios across blocks (pp. 15–16 and Figure 2), showing a clear group effect. The data cleaning procedures are also described (p. 8), and we note that no participants were excluded based on poor performance, as all were able to complete the tasks and showed learning curves.

(5) "Data analysis should take two conditions as independent variables, rather than bating success rate."

This comment appears to misunderstand the analytic strategy. Group (LI vs. CTRL) is indeed treated as a between-subjects independent variable in several analyses, including the ANOVA on batting success (p. 15). However, to test our key hypotheses about individual differences in latent inhibition, we also analyze batting success ratio as a continuous predictor, particularly within the LI group where variation in the degree of latent inhibition is meaningful (p. 17–18). This approach is theoretically motivated and statistically appropriate.

(6) "The gender, age, and other potential control variables should be considered when authors detect the difference between two group."

We did consider these variables. The groups did not differ in age or gender distribution (p. 8), and gender was included in the main ANOVA as a factor (p. 15), with no significant effects or interactions found. Thus, these variables were appropriately evaluated and ruled out as confounds.

In summary, we appreciate the reviewer’s engagement with our work. However, we respectfully submit that all six concerns are already substantively addressed in the manuscript. Repeating or emphasizing these aspects further would result in unnecessary redundancy and reduced readability. Should the editor consider that any of these points must be made more explicit in the manuscript despite our justifications here, we will be happy to revise accordingly.
