# Supplementary material for: The relationship between latent inhibition, divergent thinking, and eyewitness memory: A study on attention to irrelevant stimuli
Source: PLoS One. 2025 Jun 23;20(6):e0315158. doi: 10.1371/journal.pone.0315158 (PMC12184939; doi:10.1371/journal.pone.0315158)
Supplement: Attachment — Submitted filename: response to reviewers_.docx [file pone.0315158.s002.docx]

Dear Dr Cioffi:

We appreciate the opportunity to resubmit our manuscript titled "The Relationship between Latent Inhibition, Divergent Thinking, and Eyewitness Memory: A Study on Attention to Irrelevant Stimuli" for further consideration in PLOS ONE. Thank you and the reviewers for your work on its previous version. We have carefully addressed all the journal's requirements and have made the following revisions taking into account the reviewers' suggestions.

**Journal Requirements**

**Formatting and File Naming Compliance**:

We have revised the manuscript to ensure it meets PLOS ONE’s style requirements, including proper formatting and appropriate file naming conventions.

**Funding Statement Update**:

In accordance with PLOS ONE's guidelines, we have added a statement clarifying the role of the funders in our research.

**Copyright Issues in Figure 1**:

Due to incompatibilities between the CC-BY-SA 2.0 license from Scratch and PLOS ONE’s CC-BY 4.0 requirements, we have removed the original screenshots from the Latent Inhibition Task programmed in Scratch, as well as the images of objects from the Alternative Uses Task.

To address this, we have developed a new Figure 1, providing a schematic overview of the study's tasks, clearly illustrating the key elements and structure of each task without the use of copyrighted material.We have also updated the figure legend to clearly reflect these changes

**Changes based on reviewers’ suggestions**

**Justification for the choice of our statistical analyses and provision of data supporting this choice**

We appreciate the reviewers’ valuable feedback regarding the limited explanation of our statistical analyses. We acknowledge that the original manuscript did not fully describe the rationale for choosing the specific tests and how we verified their assumptions. In the revised manuscript, we have expanded the Methods and Results section to explicitly detail and support the analytical approach used.

**Expansion of the discussion recognizing the limitations of the study and pointing out future directions for research**

Following the recommendation of reviewer 2, we have added a new paragraph in the Discussion in which we analyze the limitations of our study and suggest avenues for future research.

**Clarification and correction of some details in the description of the Latent Inhibition Task**

We realized that the previous manuscript contained inaccuracies regarding the specific timing parameters critical for successful batting in the latent inhibition task. We previously reported the total time taken by the ball to cross the entire screen (0.3 s.) rather than the exact time from ball appearance to its arrival at the batting position (0.17 s.). Also, we did not explain clearly the relationship between the progress of the batting swing animation and the time structure of the trials. We have attempted to explain more clearly the logic of the task: the necessity for participants to respond based on the predictive auditory stimulus (the dog bark), occurring 0.4 seconds before the ball’s visual appearance, due to typical reaction times exceeding 0.17 seconds.

We have also corrected all **minor errors and typos** identified by the reviewers throughout the manuscript, thereby improving the precision and clarity of this revised version.

We believe that these revisions significantly improve the clarity, transparency and rigor of our manuscript. We thank you again for your time and work in reviewing our manuscript. We look forward to your comments.

Sincerely,

Gabriel Rodríguez

Universidad del País Vasco (UPV/EHU)

gabriel.rodriguez@ehu.eus
